# Spontaneous rupture of flexor pollicis longus tendon by tendolipomatosis in proximal phalanx: A case report

**DOI:** 10.1097/MD.0000000000012157

**Published:** 2018-09-14

**Authors:** Young-Keun Lee, Malrey Lee

**Affiliations:** a Department of Orthopedic Surgery, Research Institute of Clinical Medicine of Chonbuk National University – Biomedical Research Institute of Chonbuk National University Hospital; b The Research Center for Advanced Image and Information Technology, School of Electronics & Information Engineering, Chonbuk National University, Jeonju, Chonbuk, Republic of Korea.

**Keywords:** flexor pollicis longus, spontaneous tendon rupture, tendolipomatosis

## Abstract

**Rationale:**

Spontaneous flexor pollicis longus (FPL) tendon rupture is rarely reported. Although there are several studies investigating spontaneous FPL tendon rupture, the exact etiology of spontaneous rupture is unclear. Here, we present a case of unusual spontaneous FPL tendon rupture due to tendolipomatosis.

**Patient concerns:**

A 64-year-old right-handed retired male teacher was referred to our clinic with an inability to flex the interphalangeal joint of his left thumb.

**Diagnosis:**

Magnetic resonance imaging (MRI) revealed complete FPL tendon rupture at the level of the distal one-third of the proximal phalanx.

**Interventions:**

With the patient under general anesthesia, the FPL tendon was explored through a volar zig-zag incision. During the operation, the FPL tendon was found to be ruptured completely. Gross examination revealed a slightly yellowish denaturated tissue at the distal end of the ruptured tendon. We excised the denaturated tissue from the distal end of the ruptured tendon and sent it for histological examination. FPL tendon was repaired primarily via modified Becker method. Histopathological examination revealed normal vasculature in the tendon tissue and degenerative changes associated with lipid deposits in the tendon tissue.

**Outcomes:**

At 12-month follow-up, the patient was completely asymptomatic and had excellent IP joint range of motion (0° to 40°) in his left thumb. The wrist grip strength was 30 kg (28 kg in the Rt.) and the thumb pinch strength was 5.7 kg (4.7 kg in the Rt.). The Quick DASH score was 0.

**Lessons:**

Spontaneous rupture of the FPL tendon, attributed to degenerative changes caused by tendolipomatosis, is the first report of its kind, in the authors’ opinion. Hence we recommend to perform the histopathological examination of the debrided tissue from the ends of the ruptured tendon, if the physicians couldn’t know the exact cause of the spontaneous intratendinous rupture of the FPL. And early diagnosis followed by debridement and primary tendon repair provides an effective outcome.

## 1 Introduction

Most spontaneous intratendinous flexor tendon ruptures are reported in the flexor digitorum profundus (FDP) tendon of the ulnar 3 digits at the level of the palm, whereas it is rarely detected in the flexor pollicis longus (FPL) tendon.^[1]^ Although there are several studies investigating spontaneous FPL tendon rupture, the exact etiology of spontaneous rupture is unclear.^[2–6]^

Here, we present a case of unusual spontaneous FPL tendon rupture due to tendolipomatosis.

## 2 Consent

The patient signed informed consent for the publication of this case report and any accompanying images. Ethical approval of this study was waived by the ethics committee of Chonbuk National University Hospital because it was a case report and there were fewer than 3 patients.

## 3 Case report

A 64-year-old right-handed retired male teacher was referred to our clinic with an inability to flex the interphalangeal (IP) joint of his left thumb. One week previously, he was gardening. He had flexed his thumb around the stem of a firmly embedded weed, and as he pulled hard, he felt a sudden pop in his left thumb and found he could not flex his left thumb. Although he often played saxophone, he could not recall any specific episode of pain or discomfort within the left thumb. There was no evidence of any history of rheumatoid or other inflammatory arthritis and his rheumatoid serology was normal.

**Figure 1:**
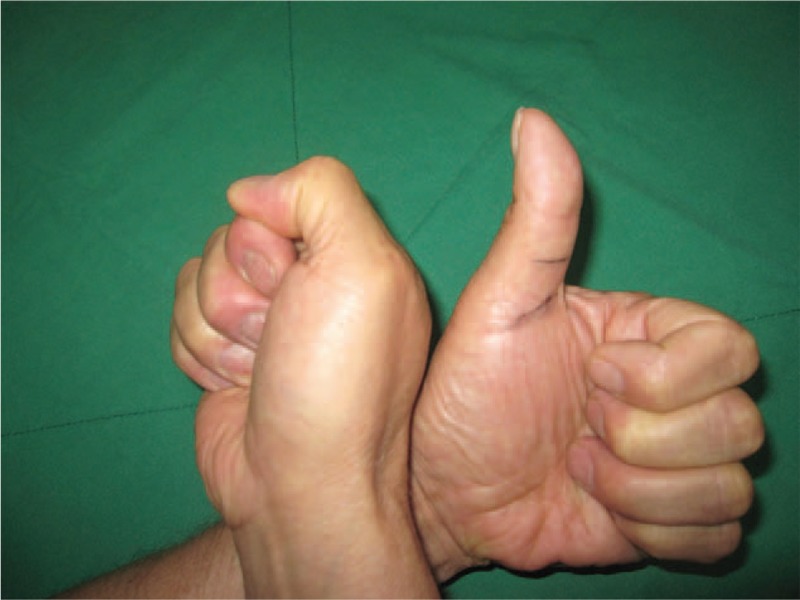
Initial image after active flexion of thumbs shows absence of flexion at IP joint in the left thumb.

Upon examination, no specific painful swelling in the left thumb was detected. However, the patient was unable to actively flex his thumb at the IP joint (Fig. 1). He had full passive range of motion of IP joint.

**Figure 2:**
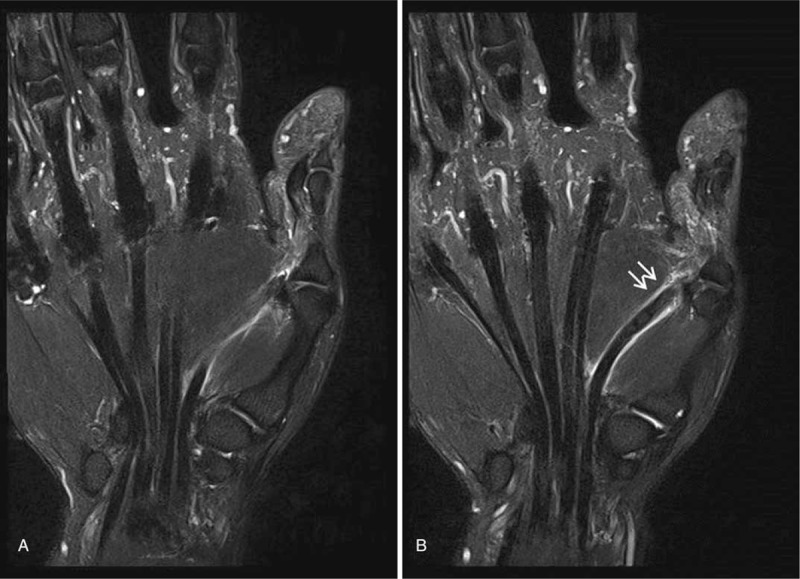
(A, B) Coronal T2-weighted MRI of the left thumb, showing complete FPL rupture at the level of the distal one-third of the proximal phalanx; the proximal end of the ruptured tendon was retracted to the level of the MP joint (arrows).

Initial plain left thumb radiographs revealed no bony abnormalities resulting in secondary tendon rupture. Magnetic resonance imaging (MRI) revealed complete FPL tendon rupture at the level of the distal one-third of the proximal phalanx and the proximal end of the ruptured tendon was retracted to the level of the metacarpophalangeal (MP) joint (Fig. 2).

**Figure 3:**
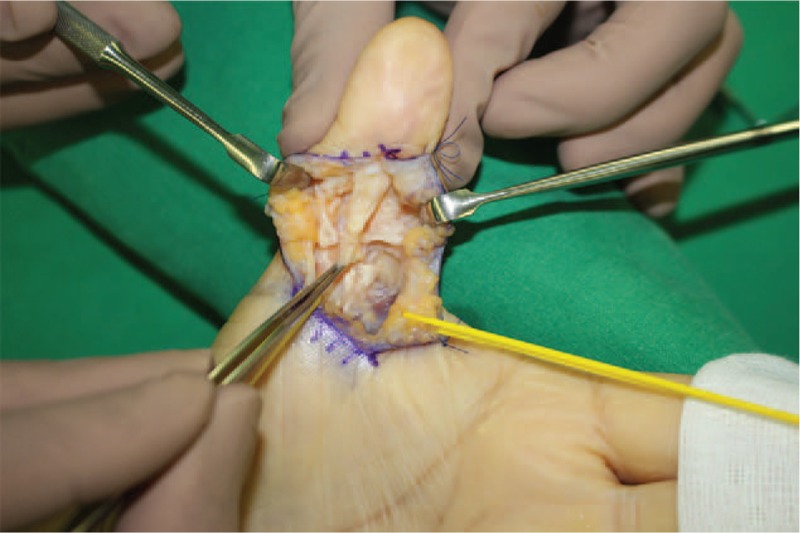
Intraoperative photograph shows complete rupture of the FPL tendon and a slightly yellowish denatured soft tissue at the distal end of the ruptured tendon.

**Figure 4:**
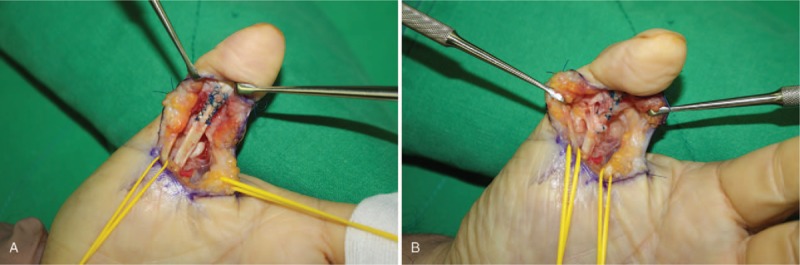
(A, B) Intraoperative photographs show (A) FPL repair with modified Becker method after denatured soft tissue is removed. (B) Oblique pulley repair.

**Figure 5:**
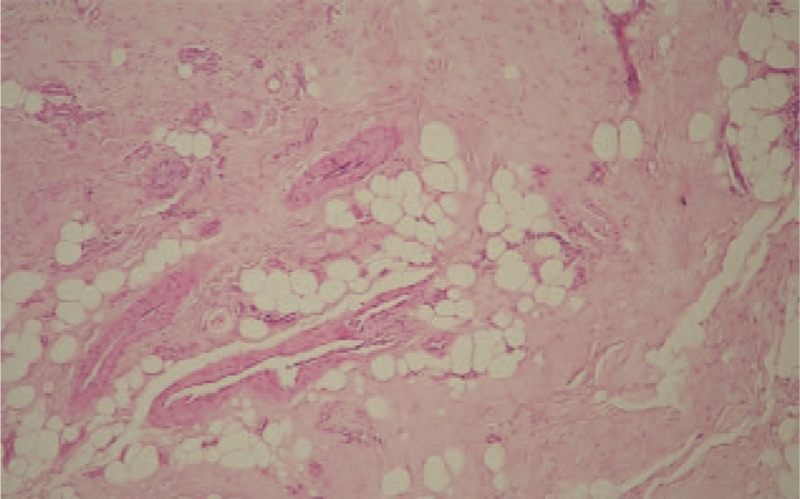
(A, B) Microscopic appearances of the denatured soft tissue from the distal end of the ruptured tendon, showing normal vascular structures in the tendon tissue and degenerative changes associated with the deposition of lipid cells in the tendon tissue (hematoxylin-eosin, original magnification ×100).

With the patient under general anesthesia, the FPL tendon was explored through a volar zig-zag incision. During the operation, the FPL tendon was found to be ruptured completely. Gross examination revealed a slightly yellowish denaturated tissue at the distal end of the ruptured tendon and the gap of the ruptured ends was interposed by a fibrous tissue (Fig. 3). No bony prominence was observed throughout the excursion of the tendon. We excised the denaturated tissue from the distal end of the ruptured tendon and sent it for histological examination. FPL tendon was repaired primarily via modified Becker method (Fig. 4). Histopathological examination revealed normal vasculature in the tendon tissue and degenerative changes associated with lipid deposits in the tendon tissue (Fig. 5).

Postoperatively, the left thumb was immobilized in a below-elbow plaster splint with extension block for 1 week, followed by dynamic splinting recommended for another 6 weeks and unrestricted full active motion at week 7.

**Figure 6:**
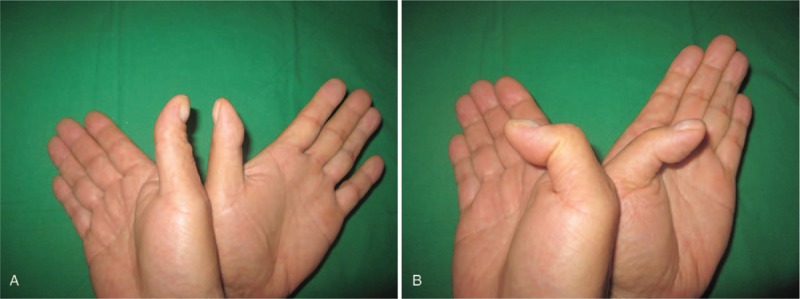
(A, B) Photographs obtained 12 months after operation show excellent IP joint ROM.

At 12-month follow-up, the patient was completely asymptomatic and had excellent IP joint range of motion (0°–40°) in his left thumb. The wrist grip strength was 30 kg (28 kg in the Rt.) and the thumb pinch strength was 5.7 kg (4.7 kg in the Rt.). The Quick DASH score was 0 (Fig. 6).

## 4 Discussion

Spontaneous intratendinous rupture is difficult to define. However, usually, it is associated with intrinsic or extrinsic pathological processes in the tendons.^[2]^ Although the etiology of spontaneous flexor tendon ruptures in the hand is unknown, repetitive impact forces sustained by the hand alone or in conjunction with tendon loading have been implicated in flexor tendon rupture.^[1]^

In 1991, Kannus and Jozsa^[7]^ evaluated tendon specimens obtained from 5 different sites after spontaneous rupture in 891 patients. They found pathological changes in all spontaneously ruptured tendons with characteristic hisopathological patterns. Most (97%) of the pathological changes were degenerative, including hypoxic degenerative tendinopathy, mucoid degeneration, tendo-lipomatosis, and calcifying tendinopathy. The most common lesion was hypoxic degenerative tendinopathy (44%).

In 1980, Hergenroede et al^[8]^ investigated the vascularity of the FPL tendon and noted that the FPL was supplied by the 4 separate vascular systems with an area approximately 10 mm in length in the relatively avascular MP joint. O’Dwyer and Jefferiss^[3]^ reported that 2 of 3 FPL tendons spontaneously ruptured in this avascular area in their case analysis. On the basis of these findings, it was suggested that the blood supply of the FPL tendon was disturbed and resulted in relative ischemia of the tendon and rupture. These findings were supported by hypoxic degenerative tendinopathy described by Kannus and Jozsa.^[7]^

However, in our case, the tendon rupture site was not in the avascular zone. However, it was observed in the distal one-third of the proximal phalanx and normal blood vessels were seen in histopathological examination, which was not due to hypoxic degenerative tendinopathy. In addition, many lipid cells were deposited in the tendon tissue, which was consistent with tendolipomatosis tendinopathy, according to Kannus and Jozsa.^[7]^ Tendolipomatosis was first described by Jozsa et al.^[9]^ Although the etiology of tendolipomatosis is unknown, similar to invasive soft tissue tumor, the lipid cells spread deep in the tendon between the collagen fibers, disrupting the integrity of the collagen eventually. Therefore, in our case, we believe that the FPL tendon rupture occurred due to a longitudinal traction force on the contracted FPL muscle that was exerted on the thumb to pull the weed out in a weakened state by tendolipomatosis. Tendolipomatosis has not been reported to occur predominantly in any particular tendon. However, it was mainly reported in the quadriceps tendon or patellar ligament, and there was no report in the FPL tendon.^[7]^

## 5 Conclusion

Spontaneous intratendinous rupture of the FPL tendon is rarely reported. Spontaneous rupture of the FPL tendon, attributed to degenerative changes caused by tendolipomatosis, is the first report of its kind, in the authors’ opinion. Hence, we recommend to perform the histopathological examination of the debrided tissue from the ends of the ruptured tendon, if the physicians could not know the exact cause of the spontaneous intratendinous rupture of the FPL. And early diagnosis followed by debridement and primary tendon repair provides an effective outcome.

## Author contributions

**Software:** Malrey Lee.

**Supervision:** Young-Keun Lee.

**Writing – original draft:** Young Keun Lee, Malrey Lee

**Writing – review & editing:** Young Keun Lee, Malrey Lee
